# Oral Warty Dyskeratoma—A Systematic Review of the Literature

**DOI:** 10.3390/diagnostics12051273

**Published:** 2022-05-20

**Authors:** A. Thirumal Raj, Kamran Habib Awan, Shankargouda Patil, Peter Morgan, Saman Warnakulasuriya

**Affiliations:** 1 Department of Oral Pathology and Microbiology, Sri Venkateswara Dental College and Hospital, Chennai 600130, India; thirumalraj666@gmail.com; 2 College of Dental Medicine, Roseman University of Health Sciences, South Jordan, UT 84095, USA; 3 Department of Maxillofacial Surgery and Diagnostic Sciences, Division of Oral Pathology, College of Dentistry, Jazan University, Jazan 45142, Saudi Arabia; 4 Head and Neck Pathology, King’s College London, Guy’s Hospital, London SE1 9RT, UK; peter.morgan@kcl.ac.uk; 5 Faculty of Dentistry, Oral & Craniofacial Sciences, King’s College London, WHO Collaborating Centre for Oral Cancer, London SE1 9RT, UK; saman.warne@kcl.ac.uk

**Keywords:** focal acantholytic dyskeratosis, oral isolated dyskeratosis follicularis, oral keratosis, oral warty dyskeratoma, oral keratosis, systematic review

## Abstract

Objective: To systematically review the clinicopathological features of oral warty keratoma based on published literature. Materials and Methods: PubMed and Scopus databases were searched for reports of oral warty dyskeratoma. Of the 52 identified articles, only 25 articles (43 cases) satisfied the selection criteria (case report/series in the English language reporting clinicopathologically diagnosed oral warty dyskeratoma/oral focal acantholytic keratosis/oral isolated dyskeratosis follicularis in humans). Risk of bias was assessed using the Joanna Briggs institute critical appraisal checklist for case reports and case series. Results: Most cases had well-circumscribed, white, nodular verruco-papillary lesions with a central depressed/crater-like area. Alveolar ridges were the most frequent sites of occurrence and tobacco was the most commonly associated risk factor. Histopathologically, the most pathognomonic feature was the supra-basal clefting. The cleft had dyskeratotic acantholytic cells (corps ronds, and grains). Below the cleft were projections of the connective tissue villi lined by basal cells. The basal cells in a few cases exhibited hyperplasia in the form of budding into the stroma, but epithelial dysplasia was not reported. The surface epithelium had crypts filled with keratin debris. Conclusion: Oral warty dyskeratoma is a rare solitary self-limiting benign entity, which due to its clinical and histopathological resemblance and associated habit history could be misdiagnosed as leukoplakia or carcinoma. None of the assessed articles provided molecular data, which in turn could be the reason for the lack of insight into the etiopathogenesis of this enigmatic lesion.

## 1. Introduction

Warty dyskeratoma represents a benign solitary tumor of the skin, vulva, and oral cavity [1]. The cause of oral warty dyskeratoma (OWK) is yet to be determined, although most cases have shown an association with tobacco smoking/chewing, local chronic trauma/irritation from a sharp tooth, or an ill-fitting denture [2]. Clinically, the lesion is largely asymptomatic, thus most cases are diagnosed during a routine oral examination. Based on the clinical appearance OWK could be classified as a verruco-papillary lesion [3]. The shape of the lesion is often nodular and well-circumscribed. Few cases have also shown a relatively smooth surface in the form of a papule, or a patch [1,2,4,5]. The color of the lesion is white, which is attributed to the hyper-keratinized state of the surface epithelium. The white color and the associated tobacco habits often lead to a provisional diagnosis of leukoplakia in case of white patch, verrucous carcinoma/verrucous hyperplasia in case of a verrucous surface, or oral squamous cell carcinoma (OSCC). An additional characteristic feature is the central depressed area of the lesion. The depressed area is often described as an ulcer, a crater, or a crypt. On palpation, the lesion appears firm which in turn is attributed to the keratin debris in these crypt/craters-like depressions of the surface epithelium. The ulcerated appearance in the background of a white verrucous/patch like lesion, often in combination with tobacco leads to a provisional diagnosis of OSCC presenting as a ulceroproliferative growth. The ulceration in the presence of a sharp cusp or an ill-fitting denture often results in a provisional diagnosis of a chronic ulcer. Histopathologically, the term focal acantholytic dyskeratosis (FAD) precisely describes OWK [1,2]. FAD presents as focal epithelial acantholysis with the acantholytic cells exhibiting varying degrees of dyskeratosis, which are often categorized as corps ronds and grains. OWK represents a small (less than 1 cm), solitary well-circumscribed lesion in the oral mucosa with histopathological features of FAD. The presence of multiple lesions, including cutaneous involvement, should prompt the consideration of Darriers disease [1]. Our objective is to systematically review the literature for the clinicopathological characteristics, treatment, and prognosis of OWK.

## 2. Materials and Methods

**Figure 1:**
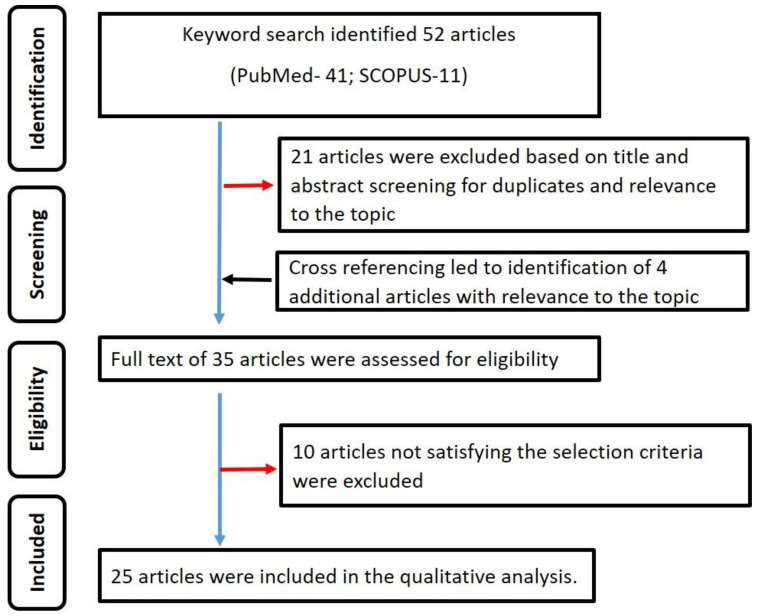
Summary of the search strategy employed in the qualitative analysis.

The International prospective register of systematic reviews (PROSPERO) was searched for systematic reviews on oral warty dyskeratoma. There were none. Thus, the present review was registered in PROSPERO (registration number: CRD42020213865). PRISMA guidelines [6,7] were strictly adhered to in the qualitative analysis (Figure 1).

The systematic review was conducted in three steps:

(1) PubMed and Scopus databases were searched using the keywords ‘oral warty keratoma; oral focal acantholytic keratosis; oral isolated dyskeratosis follicularis’. The search was done in May 2021.

(2) The identified articles were screened for potential duplicates and relevance to the topic using their titles and abstracts.

(3) The full text of the articles selected from screening was assessed using the following selection criteria:

Inclusion criteria: Case report/series in the English language reporting OWK/oral focal acantholytic keratosis/oral isolated dyskeratosis follicularis in humans. The diagnosis must have been determined using both clinical and histopathological examinations.

Exclusion criteria: Cases wherein the diagnosis lacked clinical and/or histopathological examination.

The publications satisfying the above criteria were included in the qualitative analysis. Data including the number of cases, clinical and histopathological features, the treatment rendered, and the follow-up data were extracted. Step 2 and 3 of the review were conducted by two reviewers (KHA and ATR). The inter-observer reliability in steps 2 and 3 was determined by assessing the kappa coefficient (κ).

Risk of bias assessment: The Joanna Briggs institute critical appraisal checklist for case reports and case series [8] was applied. The risk of bias was categorized as high when the study attained up to 49% score yes, moderate when the study attained 50 to 69% score, yes, and low when the study attained more than 70% score yes.

## 3. Results

A total of 52 published studies were identified. Title and abstract screening led to the exclusion of 21 articles. The remaining 31 articles were manually cross-referenced to identify further potential articles. The cross-referencing led to the identification of four articles whose titles and abstracts were relevant to the topic of interest. Based on the full-text assessment of the (31 + 4) 35 articles, only 25 articles [1,2,4,5,9,10,11,12,13,14,15,16,17,18,19,20,21,22,23,24,25,26,27,28,29] satisfied the selection criteria and were included in the systematic review. Figure 1 summarizes the search strategy used in the present review. The κ values for steps 2 and 3 of the review were 0.96 and 0.98, indicating good inter-observer reliability. Table 1 summarizes the data extracted from the included studies. A total of 43 cases were retrieved from the 25 articles [1,2,4,5,9,10,11,12,13,14,15,16,17,18,19,20,21,22,23,24,25,26,27,28,29] included in the qualitative analysis.

Study characteristics: US reported the highest number of cases (34 cases) [1,5,9,10,11,12,13,14,15,18,21,22,23,24,25,26,29], followed by UK (three cases) [4,19,20], Korea (two cases) [16,17], Australia (two cases) [28], Greece (one case) [27], and Israel (one case) [2].

Site of occurrence: The most common sites of occurrence were the masticatory mucosa including the alveolar ridge mucosa (11 cases) [1,12,21,22,23,25,28], gingiva (eight cases) [10,14,18,22,29], hard palate (seven cases) [5,11,20,22,23], palatal mucosa (three cases) [9,24,27] followed by the lining mucosa including buccal mucosa (four cases) [2,11,19,22], soft palate (three cases) [22,26], lower lip (three cases) [15,22], upper lip (two cases) [16,17], soft palate-hard palate junction (one case) [22], lateral border of the tongue (one case) [18] and the floor of the mouth (one case) [4].

Presence of potential risk factors: The most common associated risk factor was the habits including consumption of tobacco and alcohol. A total of 13 cases used tobacco [10,11,15,19,21,22,23,28], one case consumed both tobacco and alcohol [12]. Few cases had a combination of several risk factors, such as tobacco, alcohol consumption with cheek biting in one case [11]; ill-fitting denture, and tobacco usage in one case [21]. Less frequent risk factors included maxillary and mandibular denture (one case) [20], sharp tooth (one case) [2], and snuff dipping (one case) [23].

Size of the lesion: The lesion size ranged from 2 mm to 3 cm. The lesion was less than 1 cm in most (n = 22) of the cases.

Age and gender distribution of the patients: The age ranged from 33 to 81 years, with an average age of 55.7 years. Most (n = 32) of the patients were above 50 years old. Males (n = 26) were affected more often than females (n = 15). In two cases the gender of the patient was not mentioned.

Symptoms: A majority of cases did not report any symptoms from the oral lesion. Only five cases had symptoms including pain (one case) [17], mild tenderness (one case) [1], and soreness (two cases) [22,23].

Duration of lesion: Only 11 studies provided the duration of the lesion, of which seven studies reported less than 1 year. The shortest duration was 2 weeks [27], and the longest duration was 20 years [17].

Clinical diagnosis: The proximity to a sharp tooth led to the provisional diagnosis of a chronic ulcer [2]. The verrucous/pebbly appearance and firm consistency in palpation led to the provisional diagnosis of papilloma [1,22,28]. The excess keratin deposition gave the lesion a white appearance, which in turn was often diagnosed clinically as hyperkeratosis [13,18,22]. The white colour of a patch-like lesion along with an associated habit history led to the provisional diagnosis of leukoplakia [22]. A verrucous, or ulcerated lesion with an associated habit history led to the provisional diagnosis of OSCC [15,28]. One case each was diagnosed as stomatitis nicotine [22], burnt palate associated with slow-healing ulcer [22], fibroma [14], basal cell carcinoma [15], and actinic keratosis [15].

Associated lesion: One case had a history of a warty lesion on the skin of the left cheek [20]. One case had a history of OSCC, which led to mandibulectomy, followed by reconstruction of the resected mandible with a mucocutaneous flap and reconstruction plate [4]. The OWK occurred on the lateral margins of the myocutaneous flap. In one case, there was a co-existing OSCC and epidermolytic hyperkeratosis [29]. In one case, a root stump with discharging sinus was removed following which the sinus was replaced by the warty dyskeratoma [28]. One case had a co-existing smoker’s keratosis [28]. One case had co-existing mucocele with sialadenitis in the lower lip [5]. One case had a co-existing verrucous xanthoma, which was diagnosed during the histopathological examination [26].

**Figure 2:**
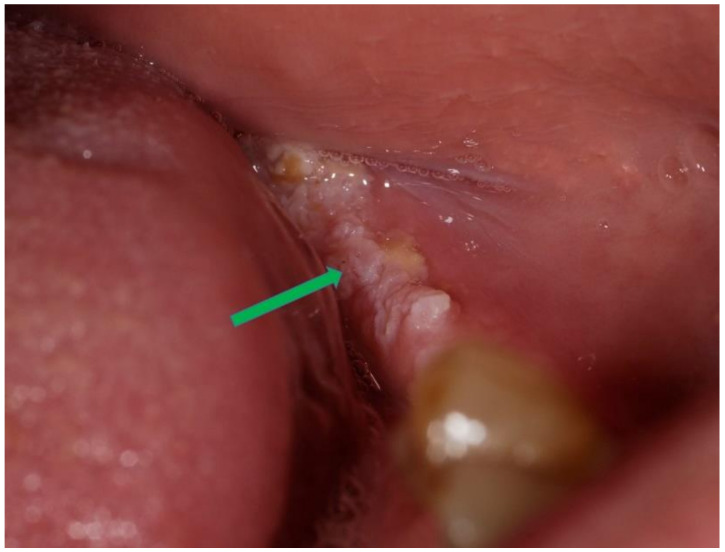
Oral warty dyskeratoma exhibiting a verrucous/warty/pebbly surface (blue arrow).

Clinical presentation: The majority of the OWK had a central depressed area which was described as ulceration, sinus opening, depressed umbilicated centre, crater, cavitation. In a few cases, the central area was described as fissures [18], and fistula [23] was used. The lesioned surface in most cases was verrucous/warty/pebbly (Figure 2).

The lesion was described as nodular to papular. The prominence of keratinization in the lesions could be evidenced by the clinical description of the lesion as a white raised lesion with a central depressed area where keratin debris could be picked off [28]. The lesion was well-circumscribed in most cases. Two cases had a white stria radiating from the central portion of the lesion [10,11]. The crusted appearance was also described in a few cases involving the lip [16,17].

**Figure 3:**
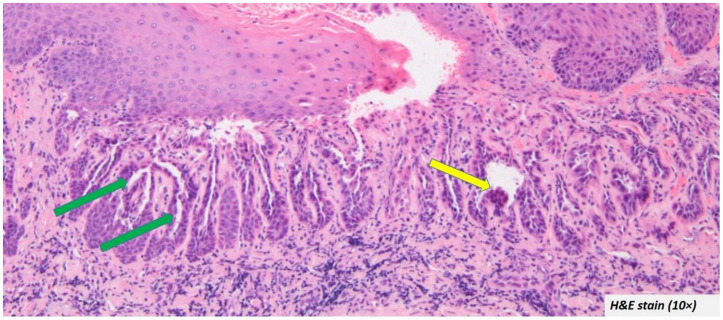
Most oral warty dyskeratoma cases included in the systematic review showed intra epithelial clefting at the supra-basal region (green arrow) and the cleft was lined by hyperplastic basal cells (yellow arrow).

Histopathological presentation: The microscopic descriptions were largely uniform. The most common and characteristic feature was the intra epithelial clefting (Figure 3—green arrow). The cleft was in most cases noted in the supra-basal region. The bottom of the cleft had connective tissue villi projections lined by a single row of basal cells which in some cases exhibited hyperplasia without atypia (Figure 3—yellow arrow).

**Figure 4:**
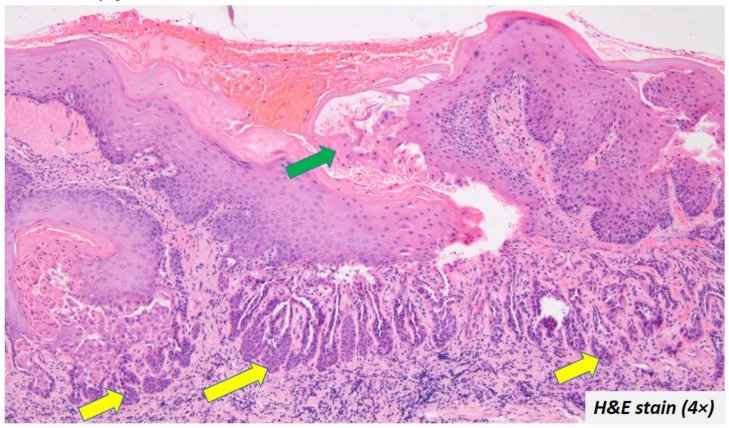
Ortho/parakeratin debris filled the depressed epithelium (green arrow) and some cases also exhibited pseudo-epithelial proliferation (yellow arrow).

The upper portion of the cleft had the spinous layer and stratum granulosum. Acanthosis was evident in some cases. The granulosum layer was prominent with large keratohyalin granules in some cases. The cleft itself contained acantholytic cells which had a varying degree of dyskeratosis. The characteristic corps ronds and grains were also described in a few cases [2,5,11,12,13,15,17,20,21,24,27,28]. The surface epithelium showed a cup-shaped depression corresponding to the areas clinically described as ulceration, sinus opening, depressed umbilicated centre, crater, and cavitation. The depressed epithelium contained ortho/parakeratin debris (Figure 4—green arrow). The pseudo-epithelial proliferation was evident in a few cases (Figure 4—yellow arrow), which had often led to the misdiagnosis of OSCC. Despite a few atypical features, the cases did not report any epithelial dysplasia.

Additional investigations: Feulgen-Schiff stain and electron microscopy examination [21,24] did not reveal any viral, parasitic, or bacterial inclusions. Immunofluorescence showed basement membrane positivity for complement 3, albumin, and fibrinogen [10].

Diagnosis: All the included cases were diagnosed as warty dyskeratoma/focal acantholytic dyskeratosis. Incisional biopsy of two cases was misdiagnosed as OSCC due to the pseudo-epithelial proliferation and dyskeratosis [18]. In those cases, the excisional biopsy revealed the characteristic features of FAD including the suprabasal clefting, keratin-filled debris in the crater of the surface epithelium, corp ronds, grains, and the lack of epithelial dysplasia.

Treatment: Excisional biopsy was the most common treatment strategy employed. In one case, an incisional biopsy was performed. Since the biopsy confirmed a diagnosis of warty dyskeratoma, the rest of the lesion was left untreated [4].

Follow up: Only nine articles provided the follow-up period, which ranged from 5 months to 14 years. No recurrence was noted in any of the reported cases.

Risk of bias assessment: Among 20 included case reports, only four case reports [2,10,13,29] provided the follow-up details. Additionally, one case report did not provide the clinical details [29]. Among the 5-case series, none provided any clear data on the consecutive or the complete inclusion of participants. Two case series [18,23] did not provide the follow-up details. Despite the above-mentioned limitations, the Joanna Briggs institute critical appraisal checklist showed that all the included case reports and series had only a low risk of bias. Table 2 and Table 3 summarize the Joanna Briggs institute critical appraisal checklist for case reports and case series, respectively.

## 4. Discussion

Warty Dyskeratoma was first reported in 1957 on the skin [30]. The first report of an OWK was issued 10 years later by Gorlin et al. [9]. OWK is a relatively less explored entity, which in turn could be largely attributed to its rare occurrence and benign self-limiting nature. Although it is a small non-progressive lesion, its clinical and histopathological resemblance to oral potentially malignant disorders (OPMDs) and OSCC often mandates additional investigation. In addition to its close appearance to a malignant entity, OWK has also been associated with major risk factors for malignancy including tobacco and alcohol [10,11,12,15,19,22,23,28]. Thus, it is necessary to understand the natural history of this enigmatic solitary lesion of the oral cavity. The present systematic review is largely based on individual case reports/case series with only one extensive published case series reporting 15 cases [22]. Given the rarity of the lesion, at present, no original studies exist analysing the varying aspects of OWK. Very few additional investigations (ultramicroscopic and immunofluorescence) are reported in the case reports/series [10,21,24]. Thus, the present systematic review is focussed largely on analysing the various clinical and histopathological features of OWK.

All the cases reported in the review were solitary. The importance of a solitary presentation lies in the close resemblance of OWK with Darriers disease, even entailing the designation of ‘pseudo-darriers disease’. Thus, in cases where the OWK-like lesions are multiple as in the present cases, Darriers disease must be considered as a potential diagnosis. OWK can be distinguished from Darriers disease by the absence of germline mutations in ATP2A2 (the gene responsible for Darier disease) and the absence of SERCA2 protein. [1,2].

As the term warty indicates, the lesion has a verruco-papillary surface in most cases. Thus, the clinical differential diagnosis often includes verrucous hyperplasia (VH), verrucous carcinoma (VC), and papillary OSCC [31]. Unlike VC, VH, and OSCC, the OWK is self-limiting with no potential for malignant transformation. In such cases, the difference in the size could be a major indicator of the nature of the lesion. Most cases of OWK are less than 1 cm, which is contrary to the progressive nature of VC, VH, and OSCC. Additionally, the OWK is well-circumscribed, which is not the case in locally invasive lesions including VC, VH, and OSCC [32]. In addition to the larger verrucous lesions, several virus-associated oral lesions are less than 1 cm in dimension. An example of such a lesion is squamous papilloma. In such cases, the presence of the viral particles in the squamous papilloma lesion would be a distinguishing factor [33]. Electron microscopic studies have confirmed the absence of viral particles in OWK, although the data are based on only two included studies [21,24]. Warty dyskeratoma from extra-oral sites has also been shown to be negative for viral (human papillomavirus) DNA [34]. Thus, although similar in presentation to most virus-induced papillary lesions, OWK may not have a viral etiology.

As OWK is considered to be a result of abnormal keratinization, all the cases presented as white lesions representing excess keratin production. Most cases present as a nodule, although a few cases of patch-like or papular lesions are also reported. A white patch-like lesion is often provisionally diagnosed as leukoplakia [22], especially with an associated tobacco history. In such cases, the absence of epithelial dysplasia, presence of suprabasal clefting, keratin-filled crypts, and the acantholytic dyskeratotic cells (corps ronds, grains) would be the distinguishing features from true tobacco-induced potentially malignant leukoplakic lesions. Few cases have presented data on consistency as ‘firm’ based on palpation [19]. The firmness can be attributed to the collection of keratin debris in the crypts of the surface epithelium. The most characteristic clinical feature of an OWK was the central areas described as depressed, crater-like, and umbilicated. This area is often filled with varying degrees of keratin debris imparting the white colour and firm consistency.

Microscopic examination reveals a series of depression and elevations in the surface epithelium which are consistent with the verruco-papillary surface of the lesion. The depression is filled with keratin debris, although overall the entire affected surface epithelium has a varying degree of hyper-ortho/parakeratosis. The characteristic histopathological feature of OWK is the cleft noted in the suprabasal region of the epithelium. The cleft is bound on its lower part by a series of projections created by connective tissue villi, which are in turn lined by a single row of basal cells. These basal cells exhibit varying degrees of hyperplasia, although atypia is not reported. The basal cell hyperplasia often leads to budding into the underlying stroma causing the appearance of pseudo-epithelial hyperplasia, which along with dyskeratosis can be misdiagnosed as OSCC [28]. In such cases, the absence of epithelial dysplasia and the presence of characteristic suprabasal clefting and keratin-filled crypts would aid in the diagnosis of focal acantholytic dyskeratosis. Within the suprabasal cleft are the dyskeratotic acantholytic cells (corps ronds and grains) [2,5,12,13,15,17,20,21,24,27,28]. Above the cleft are the thickened layer of spinosum (acanthosis) [11,12,15,16,21,23,27] and granulosum [5,20,23,28].

Although the lesions are relatively small, given their close resemblance to OPMDs and OSCC and the associated tobacco history, most cases were excised completely. None of the cases included in the present review showed any signs of recurrence or malignant transformation.

## 5. Conclusions

OWK is a rare benign solitary well-circumscribed, white verruco-papillary lesion involving the keratinized mucosa. The most characteristic clinical feature was the central depression/crater/umbilicated centre. Histopathologically, an OWK can be precisely described as a focal acantholytic dyskeratosis with varying degrees of dyskeratosis. The most characteristic histopathological feature was the supra-basal clefting, keratin-filled crypts, corps ronds, and grains. Their close resemblance to OPMDs and OSCC and the presence of associated tobacco habits mandates a histopathological confirmation of the true nature of the lesion. To date, there is no evidence of recurrence or malignant transformation in an OWK. Thus, despite its likeness to OPMDs and OSCC, an OWK is a benign self-limiting entity that, apart from a histopathological confirmation, requires no therapeutic interventions.

- OWK is a benign lesion with clinicopathological resemblance to OPMD and OSCC
- Common clinical feature includes central depression/crater/umbilicated centre
- Common histopathological features include supra-basal clefting, keratin-filled crypts, corps ronds, and grains

## Figures and Tables

**Table 1:** Summary of the data extracted from the included studies.

S. No	First Authors Name/Year of Publication/Country of Origin [Reference Number]	Number of Cases	Intra-Oral Site of Presentation		Clinical Features	Histopathological Features and Diagnosis	Treatment and Follow Up
1	Irit Allon/2012/Israel [2]	1	Buccal mucosa (opposite right maxillary second molar)		A white lesion of 0.5 cm with a verrucous surface and a small central ulceration	Epithelial proliferation was noted in the sub-mucosa. The epithelium was stratified squamous acantholytic keratinized. Multiple keratin pearls and dyskeratosis were noted. The corps ronds were present in the villi within the supra-basal split of the epithelium. Submucosa displayed a mild band of lymphocytic infiltrate. A diagnosis of focal acantholytic dyskeratosis/warty dyskeratoma was rendered.	The lesion was completely excised during the biopsy. A 5 months follow-up did not reveal any recurrence.
		Additional information: The patient was an 81-year-old female. The patients did not have any associated habits (smoking, alcohol, etc). The clinical diagnosis was chronic ulcer due to trauma (from sharp cusp).					
2	MK Basu/1978/UK [20]	1	Hard palate (left posterior portion)		A papular elevated lesion with a depressed central area	The epithelium was hyperplastic and hyperkeratinized, with a central area composed of the parakeratotic plugged cup-shaped depression. Acantholytic, dyskeratotic cells were noted between the keratotic plug and the depression Villi with cleft and corps ronds were found in the base and lateral wall of the lesion. The epithelial portion surrounding the depressed area was hypergranulotic. The sub-epithelial mucosa of the depressed area had moderate-dense inflammatory infiltrate of lymphocytes, plasma cells, histiocytes, and few eosinophils. A diagnosis of focal acantholytic dyskeratosis/warty dyskeratoma was rendered.	The lesion was completely excised during the biopsy. Follow up details were not provided.
		Additional information: The patient was a 49-year-old female. The patients had repeated warty lesions on the skin of the left cheek, which was removed by electrocautery. There was no pain or tenderness in the oral lesion. The duration of the lesion was 12 months. Patients wore complete maxillary and mandibular dentures.					
3	Gilbert Brodsky/1985/US [29]	1	Gingiva (pre-canine (papilla)		Details not mentioned	Complex intra-epithelial clefts containing dyskeratotic acantholytic cells. The epithelium was mildly parakeratotic. A diagnosis of focal acantholytic dyskeratosis/warty dyskeratoma was rendered.	The lesion was completely excised during the biopsy. Patients died 8 months later due to the OSCC recurrence.
		Additional information: The patient was a 74-year-old female. The patients did not have any associated habits (smoking, alcohol, etc). Granular mass was noted on the inter-gingival papilla from canine to the second molar. Biopsy of the granular mass revealed keratinizing OSCC. Biopsy of the pre-canine papilla also revealed a portion of the thick hyperkeratotic epithelium with granular vacuolated layer, large irregular kerato-hyaline granules, and cells with indistinct borders. This portion was diagnosed as epidermolytic hyperkeratosis.					
4	MNY Chau/1984/Australia [28]	2	1st	Upper maxillary 1st molar alveolar region	Small keratinized elliptical depression-like lesion about 1.3 × 0.9 cm dimension. From the lesional surface, keratin could be picked-off.	Due to the invasive appearance of the epithelium into the lamina propria, a histopathological diagnosis of OSCC was rendered. Examination of further sections revealed hyperplastic hyper-keratinized stratified squamous epithelium with central depression plugged with parakeratotic cells. Below the depression, there were supra-basilar clefts. Several connective tissue villi covered by a single basilar layer were noted. Above the basilar layers in the cleft, numerous corps ronds and grains were identified. Basaloid cells were hyperplastic and had extensions into the lamina propria. Dyskeratosis, abnormal mitotic figures were also noted. A mild chronic inflammatory infiltrate was noted in the lamina propria. The diagnosis was changed to oral warty dyskeratoma.	The lesion was completely excised during the biopsy. A 14 years follow-up did not reveal any recurrence.
			2nd	The alveolar ridge of the missing right 1st molar	Rectangular raised white keratotic lesion about 0.5 × 0.4 cm in dimension	Due to the appearance of epithelial dysplasia, and invasion into the lamina propria, a histopathological diagnosis of OSCC was rendered. The examination of further sections revealed acanthotic stratified squamous epithelium with numerous thin fibrovascular projections. These projections were covered by ortho-keratinized epithelium with a papillomatous surface. The stratum granulosum was thickened. The crevices between the epithelial projections were plugged with keratin and anucleated squames. Connective tissue villi in some areas were covered by just a single layer of basaloid cells which were hyperplastic and has projections in the lamina propria. One crevice had a cyst-like opening with hyper-keratotic stratified squamous epithelium and was partly filled with keratin. Dyskeratosis resembling single-cell keratinization was also found with numerous abnormal mitotic figures. A mild chronic inflammatory infiltrate was noted in the lamina propria. The diagnosis was changed to oral warty dyskeratoma.	The lesion was completely excised during the biopsy. A 6 years follow-up did not reveal any recurrence
		Additional information on the 1st patient: The patient was a 58-year-old male, who smoked 20 cigarettes per day. The patient has a root stump in the maxillary 1st molar region accompanied by a discharging sinus. The root stump was removed, after which the sinus opening was replaced by the keratinized lesion. The clinical diagnosis was papilloma, which turned out to be warty dyskeratoma. Additional information on the 2nd patient: The patient was a 51-year-old male, who smoked 20 cigarettes per day for 35 years. Smokers’ keratosis was present in the left and right retromolar region.					
5	Robert A. Danforth/1980/US [12]	1	Left mandibular ridge crest		Asymptomatic 0.4 × 0.4 cm white corrugated lesion	Hyperorthokeratotic and mildly parakeratotic stratified squamous epithelium with a central depressed area filled with the debris of epithelial cells and keratin. The depression was surrounded by a prominent granular epithelial layer. Even the spinous layer was thickened. Supra-basilar clefting with corps ronds and keratin pearls formation were noted. Connective tissue villi covered by just a single basal epithelial layer was noted. A dense chronic inflammatory infiltrate was noted in the stroma. A diagnosis of focal acantholytic dyskeratosis/warty dyskeratoma was rendered.	The lesion was completely excised during the biopsy. Follow up detail was not provided.
		Additional information: The patient was a 65-year-old male. The lesion was present for 3 months. The patient has tobacco, alcohol habits.					
6	Paul D. Freedman/1981/US [13]	1	Palatal gingiva about the left 1st molar		White raised lesion about 0.5 cm in diameter	Keratotic stratified squamous epithelium with a central depressed area filled with keratin. The spinous layer arrangement was disorganized and had dyskeratotic cells extending to the basal layer. Supra-basilar clefting was evident. Acantholytic cells were noted within these clefts. No corps ronds or grains were noted. Connective tissue villi were seen. A diagnosis of focal acantholytic dyskeratosis/warty dyskeratoma was rendered.	The lesion was completely excised during the biopsy. A 7 months follow-up did not reveal any recurrence
		Additional information: The patient was a 54-year-old female. The clinical diagnosis was hyperkeratosis.					
7	E. C. Fyfe/2002/UK [4]	1	The floor of the mouth		Reddish raised lesion of about 6 mm was seen on the floor of the mouth in the skin flaps lateral margin	Epithelium had hyper and parakeratosis. Basilar clefting with acantholytic cells and dyskeratosis was noted. Basophilic nuclei were seen in the dyskeratotic cells. A diagnosis of focal acantholytic dyskeratosis/warty dyskeratoma was rendered.	Only an incisional biopsy was made. As the diagnosis was benign, the lesion was untreated. Follow up detail was not provided.
		Additional information: The patient was a 63-year-old. Had undergone partial mandibulectomy for OSCC of the anterior floor of the mouth 2 years ago. The excised area was reconstructed using a myocutaneous flap and reconstruction plate.					
8	John L Giunta/1975/US [11]	2	1st	Buccal mucosa (opposite maxillary left canine)	A nodular, circumscribed whitish-grey, firm lesion. Its central area was depressed looking like a crater. Radiating striae were surrounding this central area.	Central depressed are filled with keratin. Multiple sub-epithelial clefts containing the acantholytic and dyskeratotic cells. The cleft base had connective tissue villi over-laid by a single row of basal cells. These basal epithelial cells showed dysplasia. The lamina propria had chronic inflammatory infiltrate (plasma cells, lymphocytes and histiocytes). A diagnosis of focal acantholytic dyskeratosis/warty dyskeratoma was rendered.	The lesion was completely excised during the biopsy. 2-year follow-up did not reveal any recurrence
			2nd	Anterior hard palate	3 mm (diameter) papular white lesion	Hyperkeratosis and acanthosis. Sub-epithelial clefts/lacunae with corps ronds and grains acantholytic and dyskeratotic cells. A diagnosis of focal acantholytic dyskeratosis/warty dyskeratoma was rendered.	The lesion was completely excised during the biopsy. 2-year follow-up did not reveal any recurrence
		Additional information on the 1st patient: The patient was a 51-year-old female. The lesion was asymptomatic. The patient had tobacco, alcohol, and cheek biting habit. She was also using an old denture. Additional information on the 2nd patient: The patient was a 33-year-old female. She had a history of cigarette smoking.					
9	Robert J. Gorlin/1967/US [9]	1	Palatal mucosa near 1st maxillary left molar		A 5 × 3 × 3 mm sessile verrucous lesion	Hyperkeratinized acanthotic stratified squamous epithelium. Central depressed area filled with a parakeratotic plug. Below this plug, several acantholytic, dyskeratotic cells were noted. Below these cells were connective tissue villi covered by a single layer of basal cells. A diagnosis of focal acantholytic dyskeratosis/warty dyskeratoma was rendered.	The lesion was completely excised during the biopsy. Follow up detail was not provided.
		Additional information: The duration of the lesion was more than 1 year.					
10	Terence Jay Harrist/1980/US [5]	1	Hard palate near left maxillary 1st molar		Verrucous hyperkeratotic lesion	Fibrovascular connective tissue spires were covered by hypergranulotic, hyperkeratotic squamous epithelium. Between these spires were depressions filled with hyper and para keratotic debris. Multiple connective tissue villi were noted in the base of these depressions. The villi were layered by basal cells showing squamous changes. Above these basal layers were the cleft containing acantholytic cells. Few corps ronds and grains were also seen. Mild lymphocytic infiltrate was noted in the underlying lamina propria. A diagnosis of focal acantholytic dyskeratosis/warty dyskeratoma was rendered.	The lesion was completely excised during the biopsy. Follow up detail was not provided.
		Additional information: The patient was a 74-year-old female. The lesion was asymptomatic. The patient also had a tender mucocele (with chronic sialadenitis) in the lower lip					
11	George E. Kaugars/1984/US [21]	1	Left retromolar area of the mandibular ridge		A white lesion with a pebbly surface about 1 × 1.5 cm	Stratified squamous epithelium with supra-basilar clefting. The Central depressed area had numerous invaginations lines with parakertin which in turn was overlayered with orthokeratin. Stratum spinosum was thickened. In the suprabasal cleft were numerous connective tissue projections lines with a single basophilic basilar layer. Numerous keratin pearls, corps ronds, and prominent granular layer were noted in the epithelium. A diagnosis of focal acantholytic dyskeratosis/warty dyskeratoma was rendered.	The lesion was completely excised during the biopsy. Follow up detail was not provided
		Additional information: The patient was a 49-year-old male. The lesion was asymptomatic. The patient was a smoker for 30 years (1 pack per day). Patients have been edentulous for 33 years. The mandibular denture was ill-fitting. The right retromolar area of the mandibular ridge also had a white leucoplakia lesion, although further details were not provided. The specimen was negative for viral inclusions as evaluated by Feulgen-Schiff stain and electron microscopy.					
12	George Laskaris/1985/Greece [27]	1	Palatal mucosa near the midline		White round 0.6 × 0.6 cm lesion with a reddish depressed center.	Hyper-orthokeratinised stratified squamous epithelium with keratin filled papillary hyperplasia and acanthosis. Suprabasal cleft with the base of the cleft having connective tissue projections layered by a single layer of basal cells. The cleft had acantholytic, dyskeratotic cells. Corps ronds and grains were also noted. A diagnosis of focal acantholytic dyskeratosis/warty dyskeratoma was rendered.	The lesion was completely excised during the biopsy. A 4-year follow-up did not reveal any recurrence
		Additional information: The patient was a 47-year-old male. The lesion was asymptomatic. No radiological changes were noted. The lesion was noted 2 weeks ago.					
13	Alan S. Leider/1984/US [18]	2	1st	The right lateral border of the tongue	Raised white nodule with a depressed umbilicated center	Thick stratified squamous epithelium with a depressed central area filled with hyperkeratotic and hyperparakeratotic plug. Subrabasilar cleft containing acantholytic, dyskeratotic cells. Basal cell hyperplasia and mild mononuclear stromal inflammatory infiltrate was noted. A diagnosis of focal acantholytic dyskeratosis/warty dyskeratoma was rendered.	The lesion was completely excised during the biopsy. Follow up detail was not provided
			2nd	Left buccal attached gingiva near mandibular 2nd premolar and 1st molar region	A white papular lesion with fissures and an erythematous border.	Papillary hyperkeratinized stratified squamous epithelium with parakeratin crypts. Suprabasal clefts with acantholytic, dyskeratotic cells. The base of the cleft has connective tissue villi lines with a single basal cell layer. A diagnosis of focal acantholytic dyskeratosis/warty dyskeratoma was rendered.	The lesion was completely excised during the biopsy. Follow up detail was not provided
		Additional information on the 1st patient: The patient was an 81-year-old male. The lesion was asymptomatic. Clinical differential diagnosis was OSCC, chronic ulcer, and granulomatous ulcer. Additional information on the 2nd patient: The patient was a 68-year-old male. The lesion was asymptomatic. The lesion has been for more than 1 year. The clinical diagnosis was hyperkeratosis					
14	Brad W. Neville/1996/US [26]	1	Soft palate (left)		2 × 2 mm papillary white lesion	Hyperplastic epithelium with a depressed central area containing parakeratin plugging. Intra-epithelial cleft (cleft was at mid-portion of the epithelium) with dyskeratotic cells. Basaloid cord-like extensions into lamina propria. These cord-like extensions showed keratin pearls. The lamina propria had foam cells (histiocyte-like cells). A diagnosis of focal acantholytic dyskeratosis/warty dyskeratoma was rendered.	The lesion was completely excised during the biopsy. Follow up detail was not provided
		Additional information: The patient was a 53-year-old male. The foam cells were CD68 positive indications of a histiocytic lineage. Due to the CD 68 positive cells, in addition to warty keratoma, an additional diagnosis of verrucous xanthoma was made. It was hypothesized that the breakdown of the epithelial cells releases lips attracting xanthoma (foam cells).					
15	J. Robert Newland/1984/US [24]	1	Palatal mucosa		Slightly elevated white well-circumscribed lesion	Parakeratinised stratified squamous epithelium with keratin filled defect extending to the spinous layer. Adjacent to the defect is suprabasal clefting containing acantholytic and corps ronds. The base of the cleft had villi lined by a single layer of basal cells. The corps ronds had abundant cytoplasm, pyknotic nuclei, and a perinuclear halo and were found at the junction between granulosum and spinosum layers. A diagnosis of focal acantholytic dyskeratosis/warty dyskeratoma was rendered.	The lesion was completely excised during the biopsy. Follow up detail was not provided
		Additional information: The patient was a 36-year-old male. The lesion was asymptomatic. The lesions were also assessed using an electron microscope.					
16	R. Patibanda/1981/US [25]	1	Mandibular alveolar ridge crest about the molar region		A 3 mm whitish elevated path with a red centre	Parakeratinized hyperplastic stratified squamous epithelium with parakeratin filed central defect. Suprabasal clefting containing acantholytic, dyskeratotic cells. Few squames and grains were also present. The base of the cleft had connective tissue villi lines by a single layer of basal cuboidal cells. A mild infiltrate consisting of chronic inflammatory cells were noted in the stroma. A diagnosis of focal acantholytic dyskeratosis/warty dyskeratoma was rendered.	The lesion was completely excised during the biopsy. Follow up detail was not provided
		Additional information: The patient was a 53-year-old male. The lesion was asymptomatic. The lesion had been there for 1 year. No radiological findings.					
17	Scott M. Peters/2017/US [1]	1	Mandibular retromolar trigone (left side)		0.4 cm verrucous/warty reddish-white papular lesion	Stratified squamous epithelium overlayered by keratotic material. Test tube-like rete ridges and suprabasal clefting. Dyskeratosis and hyperplasia of the basal cells. A slender crevice extended from the surface to the supra-basilar cleft. The crevice was filled with keratin. A diagnosis of focal acantholytic dyskeratosis/warty dyskeratoma was rendered.	The lesion was completely excised during the biopsy. Follow up detail was not provided
		Additional information: The patient was a 78-year-old male. The lesion was mildly tender on palpation. No radiological findings. Clinical differential diagnosis included exuberant granulation tissue, chronic ulcer, OSCC, and irritated papilloma.					
18	Charles E. Tomich/1971/US [23]		1st	Mandibular left alveolar ridge (buccal aspect)	The depressed area with a plug of stained material	Stratified squamous epithelium with a central depressed area filled with parakeratosis cells. Suprabasal cleft. The base of the cleft had connective tissue villi lines with cuboidal, columnar to polyhedral cells. Few of these cells had hyperchromatic pyknotic nuclei. Stroma had mild chronic inflammation. A diagnosis of focal acantholytic dyskeratosis/warty dyskeratoma was rendered.	The lesion was completely excised during the biopsy. Follow up detail was not provided
			2nd	Mandibular left alveolar ridge (distal to 2nd premolar)	White raised a 5 × 5 mm lesion with a central fistula like a tract.	Orthokeratinized stratified squamous cell epithelium with a prominent granular layer and a thickened spinous layer. An ortho and para keratin core disrupted the epithelial continuity. Suprabasal cleft with acantholytic cells. Connective tissue villi lines by columnar basal cells were noted at the base of the cleft. The basal cells had centrally or eccentrically placed nuclei. Mild chronic inflammation was seen in the stroma. A diagnosis of focal acantholytic dyskeratosis/warty dyskeratoma was rendered.	The lesion was completely excised during the biopsy. Follow up detail was not provided
			3rd	Palate (left side)	A 5 mm white lesion with a depressed crater-like central area	Hyper-orthokeratinized stratified squamous epithelium with keratin core in the center made of anucleated and parakeratin squames. Suprabasal cleft with acantholytic and dyskeratotic cells. The base of the cleft had connective tissue villi lines with columnar to spindle-shaped cells with few cells having hyperchromatic nuclei. Chronic mils stromal inflammation was noted. A diagnosis of focal acantholytic dyskeratosis/warty dyskeratoma was rendered.	The lesion was completely excised during the biopsy. Follow up detail was not provided
		Additional information on the 1st patient: The patient was a 49-year-old male. History of snuff dipping for 30 years. The snuff was placed in the left buccal vestibule. White folds were evident in the left muco-buccal fold, buccal mucosa, and the alveolar ridge. Patients were edentulous in the area of the lesion. Additional information on the 2nd patient: The patient was a 61-year-old male. He smoked 2 packs of cigarettes through adult life. Chewing caused discomfort in the lesioned area. No radiographic findings. Additional information on the 3rd patient: The patient was a 61-year-old male. The lesion caused the soreness. No radiographic findings.					
19	Susan L. Zunt/1990/US [22]	15	1st	Hard palate	2 × 3 mm nodular lesion	Features of focal acantholytic keratosis were noted in all 15 cases: Individual histological descriptions are not provided for each case. A diagnosis of focal acantholytic dyskeratosis/warty dyskeratoma was rendered.	All the cases had the lesion completely excised during the biopsy. Follow up period for each case is not mentioned, although it is mentioned that no recurrence was noted.
			2nd	Lower back buccal gingiva	Nodular lesion		
			3rd	Soft-hard palate junction (left side)	Papular lesion		
			4th	Soft palate (left side)	2 × 3 mm nodular lesion		
			5th	Lower lip (right side)	1 mm nodular lesion		
			6th	Upper back alveolar ridge (right side)	4 × 8 mm leukoplakic lesion		
			7th	Lower lip (midline)	Leukoplakic lesion		
			8th	Posterior palatal gingiva (left side)	3 × 3 mm nodular lesion		
			9th	Hard palate (right to the midline)	1 mm nodular lesion		
			10th	Lower back alveolar ridge (right side)	Nodular lesion		
			11th	Palatal gingiva near left 1st premolar	6 × 12 mm leukoplakic lesion		
			12th	Palate near left 1st and 2nd premolar	5 mm nodular lesion		
			13th	Posterior buccal mucosa (left side)	Nodular lesion		
			14th	Soft palate (right side)	5 × 5 mm leukoplakic lesion		
			15th	Lower alveolar ridge (buccal aspect, left side)	2 mm nodular lesion		
		Additional information of the 15 cases: Age of 1st to the 15th patients was 45, 49, 51, 66, 51, 52, 68, 44, 55, 62, 55, 69, 40, 66, 72 respectively. The gender of the 1st to 15th patient is F, M, M, F, M, M, M, F, F, F, M, F, F, M, unknown respectively. Duration of the lesion for the 6th, 8th, 9th, and the 13th patient were 6 months, 1 year, 6 weeks, and 2 and a half months respectively. The clinical diagnosis of the 1st to the 15th patients was stomatitis nicotina, hyperkeratosis, papilloma, leukoplakia, keratosis, hyperkeratosis/carcinoma, leukoplakia area, ulcer/raised mass, circular while raised lesion, unknown, leukoplakia strip, burnt palate associated slow-healing ulcer, unknown, leukoplakia, and hyperkeratosis, respectively. From the details collected regarding the associated symptoms for 5 patients—9th, 13th, 14th, and 15th patients were asymptomatic, while the 12th patients had soreness. Six cases had a history of using tobacco. In the remaining 9 cases, habit history was not available.					
20	Robert J. Dixey/1961/US [10]	1	Attached gingiva near the 1st molar region.		A raised white lesion with striae radiating from the lesions	Keratin-filled crypt on the surface of the epithelium. Suprabasal cleft with acantholytic, dyskeratotic cells. The base of the cleft had connective tissue villi. A diagnosis of focal acantholytic dyskeratosis/warty dyskeratoma was rendered.	The lesion was completely excised during the biopsy. A 5 months follow-up did not reveal any recurrence
		Additional information: The patient was a 36-year-old male. History of cigarette and pipe smoking for 15 years. Immunofluorescence showed basement membrane positivity for complement 3, albumin, and fibrinogen. Electron microscopy confirms the absence of parasitic, viral, and bacterial particles.					
21	Mayra L. Mesa/1984/US [14]	1	Palatal gingiva near upper right 2nd molar		Raised round white lesion	Oral mucosa with a depressed crateriform central area layered by keratin. Suprabasal cleft was noted between the keratinized epithelium and the crater base. The base of the cleft had villi projections lined by basal cells showing focal areas of hyperplasia. The cleft had dyskeratotic acantholytic cells. A diagnosis of focal acantholytic dyskeratosis/warty dyskeratoma was rendered.	The lesion was completely excised during the biopsy. Follow up detail was not provided
		Additional information: The patient was a 53-year-old female. One cigarette per day. The clinical diagnosis was a fibroma.					
22	Sung Ku Ahn/1995/Korea [17]	1	Upper lip		A 3 cm (diameter) well delineated, crusted path	Suprabasilar clefting. The cleft contained dyskeratotic cells and acantholytic keratinocytes. Few corps ronds were observed. Dense infiltration of inflammatory cells was noted in the underlying stroma. A diagnosis of focal acantholytic dyskeratosis/warty dyskeratoma was rendered.	The lesion was biopsied. Follow up detail was not provided
		Additional information: The patient was a 55-year-old female. The duration of the lesion was 20 years. The lesion was oozing and was painful. The upper lip with the lesion had atrophic changes.					
23	Ju Hee Lee/2003/Korea [16]	1	Upper lip		Crusted papules about 2–3 mm in size.	Hyper/parakeratosis, acanthosis. Suprabasilar clefting with acantholytic cells noted above the villi. Above the clefts were few dyskeratotic cells (small dark stained nuclei, and eosinophilic cytoplasm). A diagnosis of focal acantholytic dyskeratosis/warty dyskeratoma was rendered.	The lesion was biopsied. Follow up detail was not provided
		Additional information: The patient was a 41-year-old male.					
24	Brigid M. O’ Connell/1987/US [15]	1	Lower lip (right side)		5 mm papular well-delineated lesion. A crater was present in the center of the lesion.	The crater-like areas showed a depressed epidermis with acantholytic dyskeratosis. Suprabasilar clefting with acantholytic cells. Parakeratosis and dyskeratosis were present at the lesioned margins. Parakeratosis was noted without the granular layer. Corps ronds were present. Acanthosis was noted. A diagnosis of focal acantholytic dyskeratosis/warty dyskeratoma was rendered.	The lesion was completely excised during the biopsy. Follow up detail was not provided
		Additional information: The patient was a 42-year-old male. Tobacco chewer for the past 3 years. Clinical diagnosis for basal cell carcinoma, actinic keratosis, squamous cell carcinoma					
25	JC Steele/2014/UK [19]	1	Buccal mucosa (right side)		A 5 mm well-circumscribed keratotic oval-shaped lesion. Cavitation was present in the lesion.	The surface epithelium had an orthokeratin filled cup-shaped invagination. Suprabasilar clefting was noted in the epithelium adjacent to the invagination. The cleft showed grains (dyskeratotic para keratinized cells). The basal cells below the cleft showed hyperplasia. The underlying stroma had fibrosis and focal chronic inflammatory infiltrate. A diagnosis of warty dyskeratoma was made. A diagnosis of focal acantholytic dyskeratosis/warty dyskeratoma was rendered.	The lesion was completely excised during the biopsy. Follow up period for each case is not mentioned, although it is mentioned that no recurrence was noted.
		Additional information: The patient was a 60-year-old male. The duration of the lesion was 3 months. The lesion was asymptomatic. The patient had a 44-year history of smoking 20 cigarettes per day. The lesion felt fibrotic on palpation.					

**Table 2:** Risk of bias evaluation of the included studies- The Joanna Briggs institute critical appraisal checklist for case reports.

Sl. No	First Authors Name/Place of Origin/Year of Publication	1. Were the Patient’s Demographic Characteristics Clearly Described?	2. Was the Patient’s History Clearly Described and Presented as a Timeline?	3. Was the Current Clinical Condition of the Patient on Presentation Clearly Described?	4. Were Diagnostic Tests or Assessment Methods and the Results Clearly Described?	5. Was the Intervention(s) or Treatment Procedure(s) Clearly Described?	6. Was the Post-Intervention Clinical Condition Clearly Described?	7. Were Adverse Events (Harms) or Unanticipated Events Identified and Described?	8. Does the Case Report Provide Takeaway Lessons?	The Overall Risk of Bias
1	Irit Allon/2012/Israel [2]	Yes	Yes	Yes	Yes	Yes	Yes	Yes	Yes	Low
2	MK Basu/1978/UK [20]	Yes	Yes	Yes	Yes	Yes	No	Yes	Yes	Low
3	Gilbert Brodsky/1985/US [29]	Yes	Yes	No	Yes	Yes	Yes	Yes	Yes	Low
4	Robert A. Danforth/1980/US [12]	Yes	Yes	Yes	Yes	Yes	No	Yes	Yes	Low
5	Paul D. Freedman/1981/US [13]	Yes	Yes	Yes	Yes	Yes	Yes	Yes	Yes	Low
6	E. C. Fyfe/2002/UK [4]	Yes	Yes	Yes	Yes	Yes	No	Yes	Yes	Low
7	Robert J. Gorlin/1967/US [9]	No	Yes	Yes	Yes	Yes	No	Yes	Yes	Low
8	Terence Jay Harrist/1980/US [5]	Yes	Yes	Yes	Yes	Yes	No	Yes	Yes	Low
9	George E. Kaugars/1984/US [21]	Yes	Yes	Yes	Yes	Yes	No	Yes	Yes	Low
10	George Laskaris/1985/Greece [27]	Yes	Yes	Yes	Yes	Yes	No	Yes	Yes	Low
11	Brad W. Neville/1996/US [26]	Yes	Yes	Yes	Yes	Yes	No	Yes	Yes	Low
12	J. Robert Newland/1984/US [24]	Yes	Yes	Yes	Yes	Yes	No	Yes	Yes	Low
13	R. Patibanda/1981/US [25]	Yes	Yes	Yes	Yes	Yes	No	Yes	Yes	Low
14	Scott M. Peters/2017/US [1]	Yes	Yes	Yes	Yes	Yes	No	Yes	Yes	Low
15	Robert J. Dixey/1961/US [10]	Yes	Yes	Yes	Yes	Yes	Yes	Yes	Yes	Low
16	Mayra L. Mesa/1984/US [14]	Yes	Yes	Yes	Yes	Yes	No	Yes	Yes	Low
17	Sung Ku Ahn/1995/Korea [17]	Yes	Yes	Yes	Yes	Yes	No	Yes	Yes	Low
18	Ju Hee Lee/2003/Korea [16]	Yes	Yes	Yes	Yes	Yes	No	Yes	Yes	Low
19	Brigid M. O’ Connell/1987/US [15]	Yes	Yes	Yes	Yes	Yes	No	Yes	Yes	Low
20	JC Steele/2014/UK [19]	Yes	Yes	Yes	Yes	Yes	No	Yes	Yes	Low

Risk of bias categorized as high when the study attained up to 49% score yes, moderate when the study attained 50 to 69% score yes, and low when the study atttained more than 70% score yes.

**Table 3:** Risk of bias evaluation of the included studies - The Joanna Briggs institute critical appraisal checklist for case series.

S. No	First Authors Name/Place of Origin/Year of Publication	1. Were There Clear Criteria for Inclusion in the Case Series?	2. Was the Condition Measured in a Standard, Reliable Way for All Participants Included in the Case Series?	3. Were Valid Methods Used for Identification of the Condition for All Participants Included in the Case Series?	4. Did the Case Series Have Consecutive Inclusion of Participants?	5. Did the Case Series Have Complete Inclusion of Participants?	6. Was There Clear Reporting of the Demographics of the Participants in the Study?	7. Was There Clear Reporting of Clinical Information of the Participants?	8. Were the Outcomes or Follow Up Results of Cases Clearly Reported?	Was There Clear Reporting of the Presenting site(s)/Clinic(s) Demographic Information?	Was the Statistical Analysis Appropriate?	The Overall Risk of Bias
1	MNY Chau/1984/Australia [28]	Yes	Yes	Yes	Unclear	Unclear	Yes	Yes	Yes	Yes	Yes	Low
2	John L Giunta/1975/US [11]	Yes	Yes	Yes	Unclear	Unclear	Yes	Yes	Yes	Yes	Yes	Low
3	Alan S. Leider/1984/US [18]	Yes	Yes	Yes	Unclear	Unclear	Yes	Yes	No	Yes	Yes	Low
4	Charles E. Tomich/1971/US [23]	Yes	Yes	Yes	Unclear	Unclear	Yes	Yes	No	Yes	Yes	Low
5	Susan L. Zunt/1990/US [22]	Yes	Yes	Yes	Unclear	Unclear	Yes	Yes	Yes	Yes	Yes	Low

Risk of bias categorized as high when the study attained up to 49% score yes, moderate when the study attained 50 to 69% score yes, and low when the study attained more than 70% score yes.
